# Effect of Neurofeedback on Perceptual Organization, Visual and Auditory Memory in Children with Attention Deficit/Hyperactivity Disorder

**Published:** 2019

**Authors:** Abbas NESAYAN, Roghayeh ASADI GANDOMANI, Narges MOIN

**Affiliations:** 1 Department of Psychology, Faculty of Humanities, University of Bojnord, Bojnord, Iran; 2 Department of Speech Therapy, School of Rehabilitation Sciences, Iran University of Medical Sciences, Tehran, Iran

**Keywords:** Neurofeedback, Neuropsychological test, Attention-deficit/hyperactivity disorder

## Abstract

**Objectives:**

Neurofeedback is a noninvasive treatment that changes brain activity in children with attention-deficit/hyperactivity disorder and thereby improves performance in these children. We examined the effect of neurofeedback on perceptual organization, visual and auditory memory in children with attention-deficit/hyperactivity disorder.

**Materials & Methods:**

This study was quasi-experimental with pre-test, post-test design, and control group. The sample included 20 children with attention-deficit/hyperactivity disorder were selected through convenience sampling in Khorramabad, central Iran in 2017. The sample was divided into control and experimental groups. Pre-test included Rey-Osterrieth complex figure and Wechsler digit span. Rey-Osterrieth complex figure test was used to measure perceptual organization and visual memory. Wechsler digit span was used to measure auditory memory. After conducting pre-test, the experimental group participated in neurofeedback training sessions. Theta/Beta protocol was applied for all participants. The control group did not receive any intervention. Then post-test was conducted on two groups.

**Results:**

Neurofeedback training significantly improved visual memory (*P*<0.001) but neurofeedback training had no significant effect on the perceptual organization (*P*>0.05). Moreover, neurofeedback training enhanced auditory short-term memory and auditory working memory (*P*<0.05).

**Conclusion:**

Neurofeedback improved neurocognitive abilities in children with attention-deficit/hyperactivity disorder.

## Introduction

Attention deficit hyperactivity disorder is a neurodevelopmental disorder described by inattention, impulsivity, and hyperactivity. Attention deficit hyperactivity disorder frequently occur in childhood and have prevalence rate proximally 2% to 9% (1), valued to affect up to 7% of children and 5% of adult universal (2). The disorder is determined by three subtypes: predominantly inattentive type, primarily hyperactive-impulsive type and combined type (3). This condition is related to numerous negative consequences such as academic problems, behavioral disorder, social and familial disturbance (4). These children need to have academic support, retention grade and dropping out occurs among those at high levels and more are placed in special settings (2).

Authors consider damaged neurocognitive functioning as a central dysfunction of the ADHD reflected in lacks, a range of neurocognitive dysfunction such as attention, inhabitation and working memory (5). Several theories attempted to explain symptoms of attention deficit hyperactivity disorder. Functional working memory model proposes that working memory is a central deficit that underlines the attention deficit hyperactivity disorder symptoms. Hyperactivity (as a symptom of ADHD) is a compensatory function for increased cortical arousal demand for influential working memory. In these people neural areas such as frontal lobe do not work well, therefore dopamine and norepinephrine in these areas increase the motor activity (4).

Working memory deficit is related to ADHD as well as academic problems. The deficit in working memory adversely influenced processes such as focusing attention, inhabitation of unrelated stimuli, recognition the meaning of the stimulus and selecting goals. These cognitive abilities are necessary for learning (2). Working memory was assessed in children with and without ADHD. Significantly younger children with ADHD had working memory deficit (2). 

In this disorder, stimulant medication usually is recommended and assumed that it is an influential treatment of decreasing behavioral symptoms and improving neurocognitive dysfunction in children with ADHD. Nevertheless, the stimulant medication has different side effects including sleep problems, loss of appetite, and headache. Additionally, there is inadequate data about the long-term effect of this treatment (5).

Neurofeedback is noninvasive, without medicine method of brain training that can help the diversity of disorders such as pain, fatigue, depression, anxiety, sleep disorders, cognitive decline, autism, attention and hyperactivity disorder, and post-traumatic stress disorder (6,7). This method improves cognitive performance as well as regulate stress level, emotional and behavioral functioning (6). Therapists developed neurofeedback as an alternative treatment for children because they assumed that ADHD is a neurologically disorder decreasing ability of attention and behavioral control. This treatment began around 30 yr ago (8). Neurofeedback is a technique that can permit the brain to acquire self-regulation skills. These abilities change brain activity and lead to symptomatic and behavioral modifications. Throughout neurofeedback, brain performance is supervised by electrodes located on the vertex and feedback to the person by auditory and visual stimulus produced through the computer without presenting any disturbing stimulus into the brain (7). Two training protocols (theta/beta training and slow cortical potentials) are typically used in children with ADHD (8). Children with ADHD indicate increased theta (4-8Hz) and decreased beta (13-20 Hz) activity in EEG of brain activity. Increased theta and decreased beta play role in decline of attention and concentration in children with ADHD. Thus a protocol that frequently used in ADHD children cons its on, decreasing theta and increasing beta activity (5).

Since the first study that used neurofeedback treatment in ADHD in 1979, a large number studies have examined the special effects of this treatment on diverse symptoms of ADHD for example inattention, impulsivity, and hyperactivity (9). For example, the effect of neurofeedback was examined on primary symptoms of ADHD. After training of children, parents reported significant decreases in major symptoms and increased level of attention compared to control group. Additionally, neurofeedback training enhanced reaction time based on psychometric measures (8). Moreover, a meta-analysis showed large effect size for inattention and impulsivity and medium effect size for hyperactivity (9). Although a few studies examined the effect of neurofeedback on neurocognitive functions in ADHD children (10). The effect of neurofeedback was investigated on reducing post-cancer cognitive impairment in individuals with cancer. Neurofeedback training reduced the negative cognitive consequences of cancer treatment (10). 

Therefore, we explored the effect of neurofeedback training on cognitive function such as perceptual organization and working memory in ADHA children.

## Materials & Methods

This study was quasi-experimental with pre-test, post-test design, and control group. Research community consisted of all children with attention-deficit/hyperactivity disorder in Khoramabad, central Iran in 2017. The sampling method was convenience sampling and participation included 20 children with attention-deficit/hyperactivity disorder. These children were divided into control and experimental groups (10 children in each group). 

Characteristics of children included: receiving a diagnosis of attention-deficit/ hyperactivity disorder by a psychiatrist and psychologist interview, having IQ more than 85, do not have known neurological or organic diseases or developmental disorders. The mean and standard deviation for the age of children in the experimental group were M=9.7, Std. Deviation= 2.90. In the control group M=9.60, Std. Deviation= 2.45. Before implementing treatment, pre-test was conducted on experimental and control groups. Pre-tests included Rey-Osterrieth complex figure and Wechsler digit span. After conducting pre-test, the experimental group participated in neurofeedback training sessions. Training session consisted of ten sessions (30 min). After finishing the neurofeedback training sessions, post-test were implemented for the experimental and control group. 

Parental informed consent was received for all of the children participated in this study.

Collected data analyzed using SPSS ver. 19 (Chicago, IL, USA). Statistical indexes such as mean, standard deviation and Covariance analysis were used to analyze the data. 

**Neurofeedback protocol**

Each participant received ten neurofeedback sessions held 2 times a week. Each session lasted 40 min. One neurofeedback protocol (Theta/Beta) was applied to all participants. Thus they experienced similar location of electrodes that was active electrodes placed on CPz and FCz, according to the international 10/20 system. The reference electrode was located on the mastoid.

The aim of Theta/Beta protocol is to inhibit theta (4–8 Hz) and reinforce beta (13–20 Hz) activity. The signal employed in neurofeedback was very much similar to a computer game so that points are gained during three different games: a worm going toward the finish line, a monkey climbing a tree, and a face smiling. There was 30 sec break between each of these three games.

In the first 3 min of the first session, the baseline was ascertained by the trial of games using measure the Theta/Beta ratio. The participants were asked to concentrate on games. They must have reduced theta amplitude (4–8 Hz) below the baseline while increasing the beta amplitude (16–20 Hz) above the baseline.

**Rey-Osterrieth complex figure test**

Rey-Osterrieth complex figure test designed through Andre Rey in 1941 and expanded through Paul-Alex Osterrieth in 1944 (11). This test is used to assess visuospatial abilities, attention, executive function (11), perceptual organization (12), visuoconstructional abilities (13) and visual and nonverbal memory (11-13).

For doing complex figure test, first subjects are asked to copy a complex figure on the blank page in the best way. These complex figures have 18 components (11-13). Three minutes later, subjects are given another blank page and they should recall and draw the complex figure (11-13). Part one conducts for assessment of the perceptual organization and part two conducts for assessment of visual memory. 

Rey-Osterrieth complex figure test is usable in the survey of normal and atypical development in children (12). It is usually used in dementia, mild cognitive impairment, diabetes type 2, attention deficit and hyperactivity disorder and multiple sclerosis (11). 

**Digit Span subtest from Wechsler intelligence scale for children**

Wechsler digit span is an old neuropsychological test used for the measure of auditory working memory (14). This subtest is composed of two-part, digits forward and digit backward. In part one, subjects asked to listen to a digit span, then repeat it sequentially (forward). The digit spans include 3 to 9 digits and each digit span has two trails (15,16).

In part two, subjects required to listen to digits and repeat them backward. The digit spans composed of 2 to 8 digits that expressed one second each digit. Thus in each subject digit span measured for two-part (forward, backward). Factor analysis has determined that digit span forward is a task for evaluation of short-term memory and backward digit span is a task for assessment of working memory (14-16). 

## Results

According to Table 1, there was no significant difference between the average scores of perceptual organization (F= .08 & *P*=.78) between the experimental and the control groups. 

**Table 1:** Results of covariance analysis for perceptual organization variable

**Source**	**SS**	**df**	**MS**	**F**	***P***
**Pretest**	831.26	1	831.26	1.02	.000
**Group**	.06	1	.06	.08	.78
**Error**	13.73	17	.80		
**Total**	14262	20			

**Table 2:** Results of covariance analysis for visual memory variable

**Source**	**SS**	**df**	**MS**	**F**	***P***
**Pretest**	551.70	1	551.70	349.98	.000
**Group**	34.63	1	34.63	21.97	.000
**Error**	26.79	17	2.83		
**Total**	7335	20			

There was a significant difference between the average scores of visual memory (F= 21.97 & *P*=0.001) between the experimental and the control groups (Table 2).

**Table 3:** Results of covariance analysis for auditory working memory variable

**Source**	**SS**	**df**	**MS**	**F**	***P***
**Pretest**	4.32	1	4.32	8.87	.008
**Group**	14.65	1	14.65	30.09	.000
**Error**	8.27	17	.43		
**Total**	164	20			

There was a significant difference between the average scores of working memory (F= 30.09 & *P*=0.001) between the experimental and the control groups (Table 3).

**Table 4:** Results of covariance analysis for auditory short-term memory variable

**Source**	**SS**	**df**	**MS**	**F**	***P***
**Pretest**	.56	1	.56	.87	.04
**Group**	16.57	1	16.57	25.54	.000
**Error**	11.03	17	.62		
**Total**	220	20			

There was a significant difference between the average scores of short-term memory (F= 25.54 & *P*=0.001) between the experimental and the control groups.

Therefore, according to Tables 2-4 neurofeedback treatment could increase visual memory, auditory working memory and auditory short-term memory among children with attention-deficit/ hyperactivity disorder compared to the control group.

## Discussion

This study examined the effectiveness of neurofeedback training on cognitive abilities such as perceptual organization, visual memory, short-term auditory memory and auditory working memory in children with ADHD. Neurofeedback can improve visual memory, short-term auditory memory, and auditory working memory, but neurofeedback training cannot enhance perceptual organization in children with ADDH. Neuropsychological assessments show a significant difference between pretest and posttest scores after receiving neurofeedback training. 

The results of this study confirm some of the conclusions of researchers who worked on the effects of neurofeedback. The effect of neurofeedback was examined on the symptom of ADHD and neurocognitive abilities. Neurofeedback improved attention and reaction time in children with ADHD (8). In the study on neurofeedback training for children with ADHD, training was effective on behavioral and cognitive outcomes including enhancement of attention and IQ in one hand and reduced of hyperactivity and impulsivity on the other hand, which remained stable six months after training (17). The results of this study are not consistent with some studies compared the effect of neurofeedback training, physical activity and stimulant medication on neurocognitive functioning. Stimulant medication compared neurofeedback training and physical activity reduced inattention and impulsivity, in addition to enhanced inhibition (5).

A functional magnetic resonance imaging study showed neurofeedback normalized parts of brain engaging in selective attention and inhibitory control (18). In neurofeedback training sessions, children learn to increase necessary frequencies and decline the unnecessary frequencies by presenting rewards and operating conditioning thought, this process can have the impact on attention and other cognitive abilities (1). TBR neurofeedback training (increasing theta and/or reducing beta activity) is a compensatory mechanism that taught to children. Thus children could learn to be attentive because basic neural networks changed and strengthen (3). In a review symptoms in some persons were expressed with ADHD created by sleep problems (19). Neurofeedback through improvement of sleep spindle and to decrease sleep problems can progress outcomes in the individual with ADHD (19). In addition, neurofeedback was a self-regulation training that enhanced control on the brain and decreases abnormal brain activity, subsequently improve brain functions such as memory (20). 

The sample size was a potential limitation in this study and results hardly related to the population of ADHD individual. This study could be expanded to a larger sample of children with ADHD. Further studies can run on other population with special needs such as learning disabilities and examine other neurocognitive abilities such as attention and reaction time.

**In conclusion,** neurofeedback training did not make a significant difference in the perceptual organization in pre-test and post-test. One explanation for this result is that scores of the subject in pre-test of the perceptual organization was high rather than other assessments, therefore less improvement in the perceptual organization could be expected.
